# Improved success rates using videolaryngoscopy in unexperienced users: a randomized crossover study in airway manikins

**DOI:** 10.1186/s40001-017-0268-7

**Published:** 2017-08-10

**Authors:** Hendrik Eismann, Lion Sieg, Nicola Etti, Lars Friedrich, Christian Schröter, Philipp Mommsen, Christian Krettek, Christian Zeckey

**Affiliations:** 1 Department of Anaesthesiology and Intensive Care Medicine, Hannover Medical School, Carl-Neuberg-Str. 1, 30625 Hannover, Germany; 2 Trauma Department, Hannover Medical School, Carl-Neuberg-Str. 1, 30625 Hannover, Germany; 3 Department of General-, Trauma- and Reconstructive Surgery, Ludwig-Maximilians-Universität München, Marchioninistr. 15, 81377 Munich, Germany

**Keywords:** Emergency medicine, Airway management, Videolaryngoscopy

## Abstract

**Background:**

Videolaryngoscopy has been proven to be a safe procedure managing difficult airways in the hands of airway specialists. Information about the success rates in unexperienced users of videolaryngoscopy compared to conventional laryngoscopy is sparse. Therefore, we aimed to evaluate if there might be more success in securing an airway if the unexperienced provider is using a videolaryngoscope in simulated airways in a randomized manikin study. Differences between commonly used videolaryngoscopes were elucidated.

**Methods:**

A standardized hands-on workshop prior to the study was performed. For direct laryngoscopy (DL) we used a Macintosh laryngoscope, whereas for videolaryngoscopy (VL) we used the cMac, the dBlade, and a King Vision videolaryngoscope. Endotracheal intubations in three simulated normal and difficult airways were performed. Main outcome parameters were time to view and time to intubation. Cormack and Lehane (C + L) classification and the percentage of glottic opening (POGO) score were evaluated. After every intubation, the participants were asked to review the airway and the device used.

**Results:**

22 participants (14.8 ± 4.0 intubations per year, mostly trauma surgeons) with limited experience in videolaryngoscopy (mean total number of videolaryngoscopy .4 ± .2) were enrolled. We found improved C + L grades with VL in contrast to DL. We saw similar data with respect to the POGO score, where the participants achieved better visibility of the glottis with VL. The hyperangulated blade geometries of videolaryngoscopes provided a better visibility in difficult airways than the standard geometry of the Macintosh-type blade. The subjective performance of the VL devices was better in more difficult airway scenarios.

**Conclusions:**

After a short introduction and hands-on training, a videolaryngoscope seems to be safe and usable by unexperienced providers. We assume a standard geometry laryngoscope is optimal for a patient with normal anatomy, whereas VL device with a hyperangulated blade is ideal for difficult airway situations with limited mouth opening or restricted neck movement.

## Background

Proper airway management is often crucial in prehospital care of critically ill patients. There are many indications in the prehospital environment in which the patient needs a secure airway with or without the induction of general anesthesia. In the prehospital setting, the incidence of a difficult laryngoscopy varies between 1.5 and 8.0% of all intubations [1, 2]. This number is clearly higher than in in-hospital intubations—in the emergency department or in the operating room. In prehospital emergency situations one-third of intubation attempts using conventional laryngoscopes fail and have to be aborted [3]. Additionally, it has been shown that the risks of hypoxemia, aspiration and even cardiac arrest increase significantly after more than two intubation attempts [4]. A video-based laryngoscopy compared to a conventional or direct laryngoscopy shows a much higher rate of successful intubation—in particular with the first attempt [5–8].

The German Society of Anesthesiologists (DGAI) suggests completing 100 endotracheal intubations for initial training followed by at least 10 intubations per year to maintain intubation skills in the prehospital setting [9]. Unfortunately, not all physicians nor paramedics in the German Emergency Medical Service (EMS) or Helicopter Emergency Medical Service (HEMS) are able to reach these numbers as endotracheal intubation is not necessarily in the scope of many physicians’ everyday practices.

Devices for indirect or videolaryngoscopes endotracheal intubation are frequently used for the management of an unpredicted difficult airway. It is well known that difficult airways occur more frequently in prehospital care compared to in-hospital care [10].

We aim to evaluate if there might be better success in securing an airway if the unexperienced provider in the prehospital environment uses a videolaryngoscope in every first attempt of an emergency intubation.

In our study we investigated providers who are not confronted daily with airway management, which means our sample group performs less than 100 endotracheal intubations per year.

We hypothesized that with a videolaryngoscopic device the intubation success of an unexperienced provider is higher and the process is faster compared to a conventional laryngoscopy. In addition, we tried to elucidate the differences between commonly used videolaryngoscopy devices.

## Methods

We did a sample size calculation with the biometry online tools from the University of Muenster, Germany. Based on our experience in everyday clinical use of videolaryngoscopes, we hypothesized that intubation of our difficult airway manikin is 50% faster with a videolaryngoscope than with a standard laryngoscope. For an assumed level of significance of .05 and a power of 80% we needed at least 17 participants to prove our hypothesis.

Ethical approval for this study (Ethical Committee Trial ID-Number 2534-2015) was provided by the Ethics Committee of Hannover Medical School (Chairperson Prof. Dr. H. D. Tröger) on January 06, 2015.

22 participants gave written informed consent to participate in this study. The evaluated groups were trauma surgeons licensed as EMS physicians (Notarzt), pediatricians as well as EMT (Rettungssanitaeter) and paramedics (Notfallsanitaeter) (ground-EMS or HEMS), and medical students in their final year of medical school. Providers with more than 100 intubations per year were excluded from the present study.

All participants took part in a standardized workshop with a lecture about the differences between direct and video laryngoscopy followed by a hands-on workshop to familiarize our participants with the airway devices we used in the present study. None of the simulated airways (normal or difficult) were seen by the participants during this period of training. During orientation, we used unmodified Laerdal Airway Trainer (Laerdal, Germany) for training the insertion of the devices and the intubation process.

For conventional standard laryngoscopy or direct laryngoscopy (DL), we used a Macintosh laryngoscope with blade size 3 (Heine, Germany) and a LED-handle (Dahlhausen, Germany). For videolaryngoscopy (VL) we used three different devices commonly used in Germany: cMac size 4 with portable monitor (Storz Medical, Tuttlingen, Germany), dBlade with portable monitor (Storz Medical, Tuttlingen, Germany), and a King Vision videolaryngoscope with attached tube channel (Ambu, Bad Nauheim, Germany). We evaluated one device from every category: the cMac represented in our internal nomenclature the videolaryngoscopy class of video enhanced standard laryngoscopes (VESL), the dBlade represented free tube-placement videolaryngoscopes (FTV), and the King Vision is an attached tube-channel videolaryngoscope (ATV)—both the dBlade and the King Vision have a hyperangulated blade geometry as opposed to a standard Macintosh blade.

**Fig. 1:**
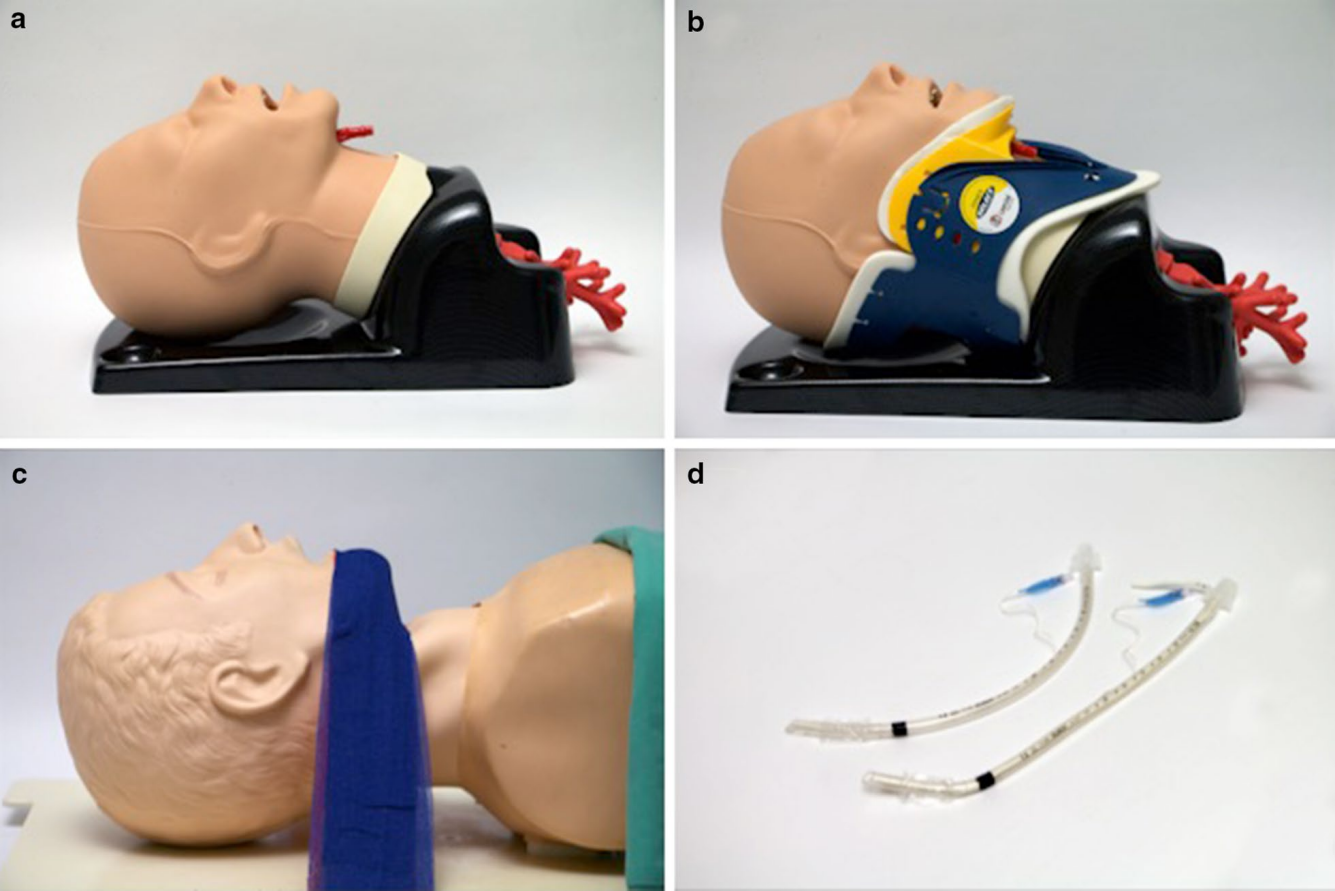
Manikins and endotracheal tubes used in the present study. **a** normal airway with free flexible mandible and cervical spine; **b** reduced mouth opening and mobility of the neck; **c** retrognathia and reduced space in the pharynx; **d** tubes used in this study.

Every intubation was performed with a 7.0 mm ID endotracheal tube (Mallinckrodt, Germany) and the intubations with laryngoscopes without tube channel (DL, VESL, FTV) with an intubation stylet were bent in straight-to-cuff-30° technique [11] (Fig. 1). During King Vision videolaryngoscopy, no stylet was used.

In our study, we used the AirSim Advanced (TruCorp, Ireland) and the Laerdal Difficult Airway Trainer (Laerdal, Norway) to simulate the normal and the two difficult airways (Fig. 1). Airway Manikin A represented a normal airway with free flexible mandible and cervical spine, Manikin B had a tight-fitted Stifneck Select collar (Laerdal Norway) in place which reduced the mouth opening and the mobility of the neck. The third manikin, C, had retrognathia and reduced space in the Pharynx. Fixating the mandible backwards as shown in Fig. 1 simulated retrognathia and decreased pharyngeal space. Elevating the base of the tongue during laryngoscopy was hindered. The tongue was inflated with 20 mbar in all three manikins to simulate its normal firmness.

We applied the El-Ganzouri index [12] to evaluate the risk of laryngoscopy on each of our manikins prior to the study. Manikin A has 1 point on the El-Ganzouri index (inter-incisor gap = 0, thyromental distance = 0, modified Mallampati class = 1, neck movement = 0, ability to prognath = 0, body weight = 0, history of difficult intubation = n/a); Manikin B has 5 points (inter-incisor gap = 1, thyromental distance = 1, modified Mallampati class = 1, neck movement = 2, ability to prognath = 0, body weight = 0, history of difficult intubation = n/a), and Manikin C has 7 points (inter-incisor gap = 1, thyromental distance = 2, modified Mallampati class = 1, neck movement = 2, ability to prognath = 1, body weight = 0, history of difficult intubation = n/a) on a scale from 0 points to 12 points. If a patient has more than 4 points on the El-Ganzouri index, the provider has to expect a difficult intubation.

Each participant in the study had to perform an endotracheal intubation on each of the three simulated airways with every device available in the study (12 intubations per participant in total). Both the order of the devices as well as the order of the manikins that had to be intubated was randomized for each participant to eliminate an effect of training by intubating the manikins in a definite order. The randomization process was performed by HE and LS.

We measured the time from when the airway device entered the mouth to the point when the glottic opening became visible (time to view) and to the intubation of the trachea (time to intubation). After the intubation, the time from sight of the vocal cords to a successful intubation was calculated (tube handling time). During video laryngoscopy, the investigators determined the point when the glottic was viewed and successful intubation—during direct laryngoscopy the participants had to indicate when the vocal cords became visible and the trachea was intubated because with a conventional laryngoscope only the provider has sight of the glottic opening. We investigated the Cormack and Lehane classification [13] and the percentage of glottic opening (POGO) score [14], too. After direct laryngoscopy, the participants were asked to independently classify their intubation using the Cormack and Lehane classification and the POGO score.

After every intubation, the participants were asked to review the airway device for the particular simulated airway using the German school grading system [1 (best) to 6 (worst)]. The intubation attempts were aborted after a maximum period of 60 s.

Individual experience in endotracheal intubation was collected via a questionnaire, which included the overall number of intubations, the number of intubations per year, and the overall number of VL intubations.

## Statistics

All collected data from the intubations attempts were processed with SPSS 23 (IBM Corporation, USA). We compared data of each airway scenario and calculated differences compared to the standard macintosh laryngoscope. Categorical data were analyzed using the McNemar test, and continuous data were analyzed using the Mann–Whitney U test. Level of significance was set at *p* < .05.

## Results

We evaluated the intubation attempts of 22 participants with a mean age of 35.7 ± 2.0 years (mean ± SEM). Several professions and disciplines were represented: 71.4% trauma surgeons, 4.8% pediatricians, 19.0% HEMS, and 4.8% EMS. Of these, 54.5% were residents, 18.2% consultants, 13.2% EMT, and 9.1% paramedics.

Five of our participating physicians (33.3%) were licensed German EMS physicians with various years of experience in prehospital settings (8.9 ± 11.8 years), whereas 11 participants (66.7%) where not yet certified. The overall experience in medicine generally was 8.8 ± 2.5 years.

In a questionnaire prior to the practical part of the study, we asked about expertise in direct laryngoscopy intubations and in videolaryngoscopic intubations. Our participants perform 14.8 ± 4.0 intubations per year. The mean total number of DL intubations was 148.8 ± 74.2 and of videolaryngoscopy it was .4 ± .2.

In the simulation phase of our study the time to glottic view and the time to intubation of the trachea were evaluated as described. The measured times and corresponding levels of significance are shown in Table 1.

**Table 1:** Intubation characteristics; intubation times

	Manikin A			Manikin B			Manikin C		
	Mean	SEM	p-value*	Mean	SEM	p-value*	Mean	SEM	p-value*
Time to view									
Direct laryngoscopy	4.6	1.0		6.6	1.1		9.5	2.7	
Storz cMac	6.2	1.0	.064	7.1	1.4	.673	10.0	2.5	.752
Storz dBlade	4.1	.5	.608	3.5	.3	.005	3.2	.3	.017
Ambu King Vision	4.2	.6	.712	4.1	.7	.039	3.6	.4	.027
Time to intubation									
Direct laryngoscopy	9.4	1.4		13.2	2.3		17.5	3.2	
Storz cMac	12.7	2.3	.090	10.6	1.9	.116	16.4	3.3	.875
Storz dBlade	13.2	3.8	.175	10.2	1.3	.229	8.5	1.5	.016
Ambu King Vision	13.7	3.1	.043	10.4	2.2	.296	11.5	1.9	.086
Tube handling time									
Direct laryngoscopy	4.6	.9		6.6	1.6		6.9	1.4	
Storz cMac	6.2	1.7	.336	4.6	1.1	.149	8.5	2.0	.682
Storz dBlade	8.7	3.4	.157	6.8	1.2	.641	5.3	1.4	.385
Ambu King Vision	9.1	2.7	.031	6.3	1.7	.898	8.0	1.9	.999

* Level of significance compared to direct laryngoscopy at the same manikin

The Cormack and Lehane classification (C + L) and the POGO score were evaluated as described above. We found improved C + L grades with videolaryngoscopy in contrast to the direct laryngoscopy. Similar data regarding the POGO score were seen, where the participants achieved a better view of the glottis with VL. The hyperangulated blade geometries provided a better view than the standard geometry of the Macintosh-type blades (Table 2).

**Table 2:** Intubation characteristics: Cormack and Lehane score, POGO score, and personal review of the participants; POGO, percentage of glottis opening; German school grading system with ratings from 1 (very good) to 6 (very poor)

	Cormack and Lehane score			POGO score			Personal review (German school grading system)		
	Mean	SEM	*p*-value*	Mean	SEM	*p*-value*	mean	SEM	*p*-value*
Manikin A									
Direct laryngoscopy	1.2	.1		77.1	3.7		2.2	.1	
Storz cMac	1.1	.1	.493	79.8	4.4	.615	2.2	.2	.803
Storz dBlade	1.0	.0	.104	91.2	2.3	.000	1.9	.3	.090
Ambu King Vision	1.0	.0	.104	93.6	2.2	.000	1.9	.2	.137
Manikin B									
Direct laryngoscopy	1.9	.2		40.2	6.3		2.7	.2	
Storz cMac	1.4	.1	.015	59.8	6.9	.012	2.1	.2	.045
Storz dBlade	1.0	.0	.000	89.1	2.4	.000	1.6	.1	.001
Ambu King Vision	1.0	.0	.000	94.3	2.2	.000	1.5	.2	.000
Manikin C									
Direct laryngoscopy	2.7	.2		12.8	4.2		4.0	.3	
Storz cMac	1.7	.1	.000	48.2	7.2	.000	2.9	.2	.000
Storz dBlade	1.1	.0	.000	84.8	2.8	.000	1.6	.2	.000
Ambu King Vision	1.1	.1	.000	83.4	4.4	.000	2.4	.3	.000

* Level of significance compared to the DL at the same manikin

The subjective performance of the individual devices was rated via the German school grading system from 1 (best) to 6 (worst). The subjective performance of the VL devices was better in more difficult airway scenarios. For a normal airway, the participants rated all devices as almost equal. In our most difficult airway, the Macintosh laryngoscope performed the worst, whereas the VL devices were rated better (Table 2).

According to this study’s design the intubation attempts were aborted after a period of 60 s. Additionally, in a post hoc analysis we calculated the number of intubations, which were successful only after 30 s, which corresponds to an increased risk of hypoxemia. We found higher success rates in DL in the normal airway (Manikin A); the devices with a different geometry compared to a standard laryngoscope (dBlade and King Vision) performed worse (100% success rate in DL; 90.5% success rate with dBlade, and 95.2% with King Vision). The data from the intubation attempts in Manikin C reveal a higher rate of successful intubations with hyperangulated blade devices (95.2% dBlade; 90.5% standard laryngoscope). After a calculated abort at 30 s, we found a larger difference between DL and dBlade (76.2% DL; 95.2% dBlade). We also found more successful intubations with dBlade versus King Vision—however the McNemar test revealed no significant difference (Table 3).

**Table 3:** Intubation characteristics; intubation success

	Intubation success—abort after 30 s**			Intubation success—abort after 60 s		
	Frequency	Percent	*p*-value*	Frequency	Percent	*p*-value*
Abort after 60 s						
Manikin A						
Direct laryngoscopy	21	100		21	100	
Storz cMac	19	90	***	21	100	***
Storz dBlade	17	81.0	***	19	90.5	***
Ambu King Vision	18	85.7	***	20	95.2	***
Manikin B						
Direct laryngoscopy	19	86.4		22	100	
Storz cMac	20	90.9	1.000	21	95.5	***
Storz dBlade	21	95.5	.500	21	95.5	***
Ambu King Vision	18	85.7	1.000	20	95.2	***
Manikin C						
Direct laryngoscopy	16	76.2		19	90.5	
Storz cMac	16	76.2	1.000	20	95.2	1.000
Storz dBlade	20	95.2	.125	20	95.2	1.000
Ambu King Vision	16	84.2	1.000	16	84.2	1.000

* Level of significance compared to direct laryngoscopy at the same manikin. McNemar test

** Post hoc analysis of a theoretical abort after 30 s

*** Processing of a McNemar test was not feasible due to constant values in direct laryngoscopy

## Discussion

Skillful airway management in critically ill or injured patients is decisive for clinical outcome [15]. Since 1943 direct laryngoscopy with a Macintosh laryngoscope described by Sir Robert R. Macintosh has been used as a quasi-standard for endotracheal intubation [16]. However, it has been proven that successful direct laryngoscopy requires a lot of training and experience. In this context, completing up to 100 intubations in a clinical setting prior to the first prehospital airway-management case has been proposed by different medical societies. Furthermore, certain annual quotas of intubations are needed to maintain airway management skills. Unfortunately, many providers cannot achieve these numbers. There might be an advantage in using a videolaryngoscope instead of the Macintosh laryngoscope in the prehospital setting. A benefit of the videolaryngoscope for novice emergency physicians in the setting of an emergency department has previously been described [5–7, 17, 18].

We aim to evaluate if there might be better success in securing an airway if the unexperienced provider in the prehospital environment uses a videolaryngoscope in every first attempt of an emergency intubation.

In our study, all participants were able to operate all of the provided videolaryngoscopes after a short theoretical lecture on the background of indirect laryngoscopy followed by a short training period with manikins. Moderate training, therefore, seems adequate to introduce a videolaryngoscopy device to providers and for them to operate it safely. We recognized a steep learning curve in our free hands-on exercises.

Three different modified airway manikins were provided to simulate three grades of difficulty in prehospital emergency medicine. We saw little difference during the intubation attempts on our normal airway manikin (Manikin A) and there was no difference in the overall intubation time. The success rate for the unmodified airway manikin was higher during intubation with Macintosh style blades compared to devices with hyperangulated geometry. In addition, we found significantly better visibility of the glottic opening measured via POGO score with the FTV and ATV devices. It can be assumed that the classical direct laryngoscopy performs better in patients with normal anatomy. One reason might be that all of our participants have had training with DL and with the optimal positioning of the patient. All our participants were familiar with the alignment of the oral, pharyngeal, and laryngeal axis. There was almost no training with VL devices at all. These results were also confirmed by the study of Szarpak et al. In their study investigating novice physicians and their performance on endotracheal intubation in a simulation setting using either the Macintosh or the IntuBrite videolaryngoscope, no superior effect of the videolaryngoscope was shown. In addition, the efficacy of the first intubation attempt was higher for the Macintosh [19].

A limitation of the present study is that we did not investigate the force that was used during intubation with the Macintosh laryngoscope. Maassen and Lee showed that less force is applied to the maxillary incisors during videolaryngoscopy compared to classic laryngoscopes [20, 21]. In addition, stylet requirement was significantly reduced by using videolaryngoscopy [22]. This was also shown in morbidly obese patients [23]. In addition, Raymondos et al. [24] found that videolaryngoscopes with hyperangulated blades need less force for intubation in difficult airways compared to Macintosh shaped laryngoscope blades. Consecutively, less peri-intubation complications due to extensive forces can be expected with VL devices.

The first difficult airway had an immobile cervical spine and a restricted mouth opening. We observed a significantly shorter time-to-view of the glottic opening with the dBlade compared to the standard laryngoscope but, as described above, we did not find a significant difference in the overall intubation time. The dBlade videolaryngoscope has in addition to its hyperangulated shape a reduced thickness compared to standard geometry blades and seems to be superior in airway situations with restricted mouth opening. In many difficult airways, it is essential to achieve a fast and extensive view of the vocal cords to optimize the intubation attempt. Regarding the success rate, we found no significant difference depending on the use of DL or the different VL devices. However, there was a significant improvement in the visibility of the glottis—higher POGO scores and lower C + L classifications were reported. In general, it seems conceivable that practitioners not performing intubation procedures on a routine basis might find help by using the video laryngoscope since they are not aware of or routinely do not use the accurate sniffing position. In addition, trainees tend to hyperextend the neck, displacing the larynx anteriorly and fail to perform intubation using a Macintosh blade. McElwain et al. [25] showed in a collective of experienced operators that the VLs ability to “look around the corner” improves its view to the glottis in patients with immobile c-spine. The alignment of the oral, pharyngeal, and laryngeal axis to achieve the best possible view to the cords in direct laryngoscopy is not feasible. The overall subjective satisfaction of the participants was increased for all three videolaryngoscopes.

From the authors’ point of view, the third simulated airway represents the most difficult one due to the reduced pharyngeal space and the fixated mandible. In this simulated airway, significantly reduced times-to-view were found using the dBlade and the King Vision device. Both devices with their hyperangulated blade geometry appear to be an optimal configuration for an airway with limited mouth opening and retrognathia. The intubation time was significantly reduced in comparison to the Macintosh laryngoscope. Furthermore, higher success rates with hyperangulated blades could be observed at the 30 and 60 s cut off points. The visibility of the glottis (Cormack and Lehane score, as well as POGO score) was significantly better, and the participant reviews of the VL devices were also significantly improved. The common problem of dental damage during intubation correlates with the applied force to the patients’ teeth. Hyperangulated blades reduce the force applied to the teeth, leading to reduced dental damage during intubation [21, 24]. We presume hyperangulated videolaryngoscopes to be safer and faster for securing difficult airways, similar to our modified manikins. In summary, the Macintosh laryngoscope performed best in a normal airway. In contrast, VL devices from the FTV and the ATV groups performed best in our difficult airway scenarios. Based on these findings, we consider an assessment of the patient’s airway crucial to differentiate between normal anatomy and difficult airway. One approach to identify a difficult airway is the use of a mnemonic such as the LEMON mnemonic [26] or the El-Ganzouri Index [12]. In this context, Truszewski et al. [27] performed a randomized cadaver study investigating the ability of paramedics to perform endotracheal intubation during continuous chest compressions. The investigators were able to demonstrate improved intubation rates and a shorter intubation time using the Pentax AWS videolaryngoscope compared to the Macintosh laryngoscope.

The present study is substantially limited by the use of a manikin-based design. Thus, performance of all videolaryngoscopes is limited if blood secretion impedes the view through the lens, which was not simulated in our study. This issue demands the possibility and the ability to use a standard geometry blade as a rescue tool if the device’s camera is contaminated with secretions. Another option is to use a VESL device and to switch to direct laryngoscopy if the camera is malfunctioning.

Another factor is that the limited anatomic reproducibility of our airway manikins compared to humans-simulated airway scenarios cannot entirely reflect the individual variability of upper airway anatomy in human patients [28]. Therefore, further investigations are needed in order to transfer our findings into everyday clinical practice. Furthermore, the potential need for induction of general anesthesia was excluded, and we also disregarded the exact, but inter-individual variable oxygen reserve during intubation attempts by limiting the intubation time to 60 and to 30 s [29]. In the study at hand, a heterogeneous study population was investigated. In upcoming studies, this should be addressed by analyzing the specific groups with different theoretical backgrounds.

For excellently trained providers such as anesthetists, there seems to be little benefit regarding success rate and intubation time of a videolaryngoscope over a standard laryngoscope. Some kind of mental “Macintosh-blockade” through daily practice with direct laryngoscopy devices and limited use of VL could be assumed. As described earlier, a reduced complication rate must be assumed with using of VL. For the unexperienced provider, a hyperangulated videolaryngoscope could be the device of choice for emergency intubations.

In the literature, each fifth emergency intubation using a videolaryngoscope has been described as not successful [30–32]. However, experience level of the study participants was not clearly described which might cause bias. After our standardized workshop and hands-on training prior to the intubation attempts, we could not confirm these findings in our standardized simulated environment. Despite the limited introduction to and training with VL devices we found excellent success rates with devices, which were new to the participants. Our data support the implementation of videolaryngoscopy in the preclinical setting with its nature of oftentimes moderately trained staff in airway management.

## Conclusion

After a short introduction and limited hands-on training, a videolaryngoscope seems to be safe and usable by providers, primarily unexperienced in the use of VL. We assume a standard geometry laryngoscope is optimal for a patient with normal anatomy and a VL device with a hyperangulated blade is ideal for difficult airway situations with limited mouth opening or restricted neck movement. The assessment of the patient’s airway is important prior to the induction of any general anesthesia. However, obtaining skills in direct laryngoscopy in addition to videolaryngoscopy is crucial if VL’s electrical parts and camera fail.
